# EPB41L3, TSP-1 and RASSF2 as new clinically relevant prognostic biomarkers in diffuse gliomas

**DOI:** 10.18632/oncotarget.2745

**Published:** 2015-01-30

**Authors:** Noemi Perez-Janices, Idoia Blanco-Luquin, Maria Teresa Tuñón, Edurne Barba-Ramos, Berta Ibáñez, Idoya Zazpe-Cenoz, Maria Teresa Martinez-Aguillo, Berta Hernandez, Enrique Martínez-Lopez, Agustin F. Fernández, Maria Roasario Mercado, Teresa Cabada, David Escors, Diego Megias, David Guerrero-Setas

**Affiliations:** ^1^ Cancer Epigenetics Group, Navarrabiomed-Fundación Miguel Servet, Navarra, Spain; ^2^ Department of Pathology Section A, Complejo Hospitalario de Navarra, Navarra Health Service, Navarra, Spain; ^3^ Navarrabiomed-Fundación Miguel Servet, Navarra, Spain; ^4^ Red de Evaluación en Servicios Sanitarios y Enfermedades Crónicas (REDISSEC), Navarra, Spain; ^5^ Department of Neurosurgery, Complejo Hospitalario de Navarra, Navarra Health Service, Navarra, Spain; ^6^ Department of Medical Oncology, Complejo Hospitalario de Navarra, Navarra Health Service, Navarra, Spain; ^7^ Department of Radiation Oncology, Complejo Hospitalario de Navarra, Navarra Health Service, Navarra, Spain; ^8^ Cancer Epigenetics Laboratory, Instituto Universitario de Oncología del Principado de Asturias (IUOPA), HUCA, Universidad de Oviedo, Asturias, Spain; ^9^ Department of Radiology, Complejo Hospitalario de Navarra, Navarra Health Service, Navarra, Spain; ^10^ Confocal Microscopy Core Unit, Spanish National Cancer Research Centre, Madrid, Spain

**Keywords:** Brain tumors, EPB41L3, DNA methylation, prognosis, RASSF2, TSP-1

## Abstract

Hypermethylation of tumor suppressor genes is one of the hallmarks in the progression of brain tumors. Our objectives were to analyze the presence of the hypermethylation of *EPB41L3*, *RASSF2* and *TSP-1* genes in 132 diffuse gliomas (astrocytic and oligodendroglial tumors) and in 10 cases of normal brain, and to establish their association with the patients’ clinicopathological characteristics. Gene hypermethylation was analyzed by methylation-specific-PCR and confirmed by pyrosequencing (for *EPB41L3* and *TSP-1*) and bisulfite-sequencing (for *RASSF2*). *EPB41L3*, *RASSF2* and *TSP-1* genes were hypermethylated only in tumors (29%, 10.6%, and 50%, respectively), confirming their cancer-specific role. Treatment of cells with the DNA-demethylating-agent 5-aza-2′-deoxycytidine restores their transcription, as confirmed by quantitative-reverse-transcription-PCR and immunofluorescence. Immunohistochemistry for *EPB41L3*, *RASSF2* and *TSP-1* was performed to analyze protein expression; p53, ki-67, and CD31 expression and *1p/19q* co-deletion were considered to better characterize the tumors. *EPB41L3* and *TSP-1* hypermethylation was associated with worse (*p* = 0.047) and better (*p* = 0.037) prognosis, respectively. This observation was confirmed after adjusting the results for age and tumor grade, the role of TSP-1 being most pronounced in oligodendrogliomas (*p* = 0.001). We conclude that *EPB41L3, RASSF2* and *TSP-1* genes are involved in the pathogenesis of diffuse gliomas, and that *EPB41L3* and *TSP-1* hypermethylation are of prognostic significance.

## INTRODUCTION

Brain tumors are heterogeneous cancers that comprise various tumor subtypes classified by the state of differentiation of glial progenitors, including astrocytomas and oligodendrogliomas (ODs). Glioblastoma multiforme (GBM) is the most common primary brain tumor and the most aggressive diffuse glioma in adulthood [1]. GBM is usually fatal, and is classified as a grade IV tumor by the World Health Organization (WHO). GBM tumors are characterized by a 5-year survival rate of 4.7%, with frequent recurrences after the standard treatment, which consists of maximum surgical resection, followed by radiation with concurrent and adjuvant chemotherapy [2]. This recurrence is often caused by the appearance of resistance to standard oncological treatments and regrowth from invasion zones beyond the resection margins. ODs are more differentiated neoplasms (grade II) with better prognosis that invariably progress to higher-grade tumors, such as anaplastic ODs [3].

Molecular alterations are in part responsible for the malignancy and recurrence of primary brain tumors. Thus, the search for new biomarkers of prognostic value is essential for a better understanding of the pathophysiology of these diseases [4], with examples such as *MGMT* hypermethylation in GBM [5]. No studies have addressed the presence or the prognostic role of *EPB41L3* and *RASSF2* hypermethylation in diffuse gliomas, and there have been few studies of *TSP-1*; the hypermethylation of these genes is known to be clinically significant in other types of cancers [6–8].

The *EPB41L3* gene (*DAL-1/4.1B*: *differentially expressed in adenocarcinoma of the lung*) is located in the *18p11.3* region and codes for an adhesion protein belonging to the 4.1 family of membrane-associated proteins, which is strongly expressed in the brain [9, 10] and also regulates cell growth [11]. The *EPB41L3* gene is a suppressor gene that is altered in meningiomas [12], and in GBM, where it contributes to tumor outgrowth [13]. The mechanism of epigenetic inactivation of the *EPB41L3* promoter has been described in several types of carcinoma, including nasal NK/T-cell lymphoma and non-small cell lung carcinoma [14, 15], but it has not previously been explored in brain tumors.

Ras signaling contributes to the activation of the mitogen-activated protein kinase/extracellular signal-regulated kinase (MAPK/ERK) cascades that are involved in cancer progression [16]. The Ras association (RalGDS/AF-6) domain family member 2 (RASSF2) protein belongs to the RASSF family that participates in Ras signaling. The *RASSF2* gene resides at *20p13* and its product is strongly expressed in brain [17, 18]. The members of the *RASSF* gene family are more frequently inactivated by hypermethylation than by mutations, as previously described in the better-known *RASSF1* gene [19]. The RASSF2 protein exhibits suppressor functions as it interacts with the proapoptotic MST kinases, which are known to activate the SAPK-JNK signaling pathway, leading to subsequent apoptosis [20]. The inactivation of this gene by hypermethylation has been described in several types of cancer [7, 21–26], but it is not known whether it is present in GBM or OD.

Increased mitogenic signaling and angiogenesis drive malignant glioma growth [27]. The *TSP-1* gene encodes a large oligomeric extracellular matrix (ECM) protein that mediates cell–cell and cell–matrix interactions by binding to other ECM proteins, and that participates in cell attachment, proliferation, differentiation and apoptosis, and protects against angiogenesis [28, 29]. Nevertheless, this protective role in angiogenesis was not found in metastatic prostate cancer that displayed increased TSP-1 expression [30]. The role its silencing by hypermethylation may play has not previously been studied in brain tumors.

Our objectives were to analyze the presence of methylation in the *EPB41L3*, *RASSF2* and *TSP-1* genes in brain tumors and its prognostic role in patients. The treatment of cell lines with the demethylating agent 5-aza-2′-deoxycytidine (5-aza-2′dC) and the histone deacetylase inhibitor trichostatin A (TSA) was also studied to examine the restoration of the mRNA expression of these genes.

## RESULTS

### Analysis of methylation of EPB41L3, RASSF2 and TSP-1 genes

*EPB41L3* and *RASSF2* were hypermethylated in all cell lines, whereas *TSP-1* was hypermethylated in all except for the U87MG line. The levels of methylation varied from 65 to 100% (data not shown).

**Figure 1:**
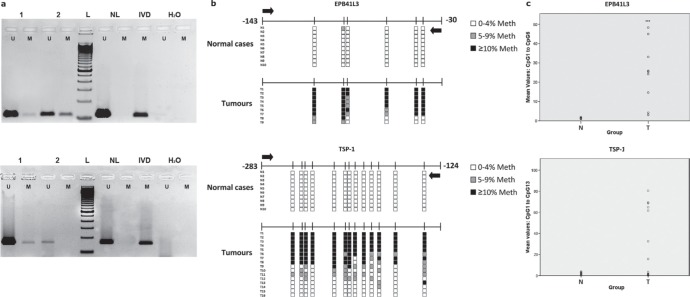
**(a) Methylation-specific PCR (MSP) of *EPB41L3* (upper) and *TSP-1* (lower) genes**. In the case of *EPB41L3,* cases 1 and 2 are less and more methylated, respectively; for *TSP-1*, case 1 is methylated and case 2 is unmethylated (U: unmethylated; M: methylated; NL: negative control; IVD: *in vitro-*methylated DNA). **(b)** Pyrosequencing results of *EPB41L3* (upper) and *TSP-1* (lower) analysis in normal (N) and tumoral (T) cases. The diagram shows the percentage methylation of the CpG positions (vertical lines) in each normal and tumoral case, represented by squares. Black arrows represent the location of the MSP primers. **(c)** Mean DNA methylation values of *EPB41L3* (upper) and *TSP-1* (lower) in normal (N) and tumoral (T) cases.

All three genes were hypermethylated to varying degrees in the tumors (Table 1, Figure 1a, Supplementary Figure 1). *TSP-1* and *RASSF2* genes had the highest (50.0%) and lowest (10.6%) frequencies of hypermethylation, respectively. All genes were always unmethylated in non-cancerous tissue from healthy individuals, as indicated by the absence of a DNA band from the unmethylated reaction. The percentage DNA methylation of these genes was not associated with the age of the patients (data not shown). These results were confirmed by PSQ (*EPB41L3* and *TSP-1*) in 10 and 16 tumors in the cases of *EPB41L3* and *TSP-1*, respectively (Figure 1b). It was possible to analyze all the cases considered in this confirmation except for the *EPB41L3* gene in one tumoral case. The mean (and SD) methylation percentages at all the CpG positions in the tumors were 41.02 (29.09) and 25.25 (19.9) for the *EPB41L3* and *TSP-1* genes, respectively (Figure 1c). The extent of methylation of *EPB41L3* and *TSP-1* in the tissue of healthy subjects for *EPB41L3* and *TSP-1* (10 cases) was less than 3% and 2%, respectively; thus, the means (and SDs) for normal tissue for the two genes were 1.36 (0.32) and 0.79 (0.31), respectively.

**Table 1:** Demographic, pathological and molecular data of patients

*Demographic data*	
**Median age** (range)	58.5 (46.0–66.0)
**Sex**	
Men	84 (64%)
Women	48 (36%)
***Pathological characteristics***	
**Tumour resection**	
Gross total	26 (21%)
Subtotal	68 (55%)
Partial	12 (10%)
Biopsy	17 (14%)
**Tumour grade^1^**	
I	9 (7%)
II	14 (10%)
III	26 (20%)
IV	83 (63%)
**Tumour lineage**	
Astrocytic	96 (73%)
Oligodendroglial (OD)	29 (22%)
Astrocytic with OD differentiation	7 (5%)
***Ki-67* grade**	
Low	60 (47%)
Intermediate	51 (40%)
High	16 (13%)
***P53* expression**	
Low-Intermediate (≤ 60% cells)	97 (77.6%)
High (> 60% cells)	28 (22.4%)
***Gene methylation***	
***EPB41L3***	
Yes	29 (29%)
No	71 (71%)
***RASSF2***	
Yes	10 (10.6%)
No	84 (89.4%)
***TSP-1***	
Yes	58 (50%)
No	57 (50%)

1 Grade according to the World Health Organization (WHO) grading system

The methylation of RASSF2 in 10 tumors and the non-methylation in 10 normal cases, as determined by MSP, were confirmed by BS (Supplementary Figure 1). *RASSF2* was not hypermethylated in any of the clones obtained from normal brain tissue (Supplementary Figure 1) but was hypermethylated in 51 to 78% of tumoral cases.

The percentages obtained with these techniques were not identical because of their different sensitivities [35, 36], but there was a clear association between the MSP and PSQ/BS results. Thus, in the case of *EPB41L3* and *TSP-1* the difference between the values obtained by MSP and PSQ was significant (*p* < 0.001 and *p* = 0.035, respectively), and MSP and BS results were also associated in the case of *RASSF2* (*p* < 0.001). *TSP-1* hypermethylation was more common in patients who had previously had cancers (*p* = 0.034), and in grades I-II compared with advanced grades III-IV (though this relationship was not statistically significant; *p* = 0.160). No other associations between molecular and pathological variables were found.

### Analysis of EPB41L3, RASSF2 and TSP-1 transcription by qRT-PCR in control and treated cell lines

**Figure 2:**
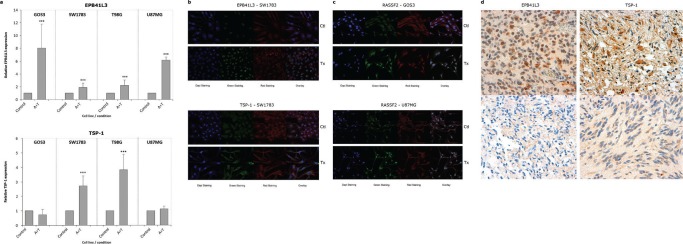
**(a) Bar graphs showing the relative level of EPB41L3 and TSP-1 expression, respectively, in treated cell lines (A+T) compared with control cells analyzed by qRT-PCR, using GAPDH expression as a normalizer**. Bars represent the mean ± standard deviation of the relative expression level of three experiments. The value obtained for untreated cells was taken as 100%. *** indicates statistically highly significant (*p* < 0.01) differences in gene expression. Immunofluorescence images of EPB41L3 (b, upper), TSP-1 (b, lower) and *RASSF2* **(c)** in cell lines (x630). The target protein is indicated with Alexa Fluor 488 (green staining), and the cytoskeleton was marked with phalloidin. An increase in the expression of the EPB41L3 and TSP-1 proteins is observed in the cytoplasm of the SW1783 cells; in the case of RASSF2 the increase of expression in the cytoplasm and nucleus of GOS3 and U87MG is shown. IHC staining for a tumor positive (d, upper left), and negative or weak (d, lower left) for *EPB41L3* expression, unmethylated and methylated for this gene, respectively (x600). A tumor positive (d, upper right), and negative or with weak (d, lower right) expression of *TSP-1*, unmethylated and methylated for this gene, respectively (x600).

Relative expression levels per gene and cell line are shown in Table 2. There were 8- and 6-fold increases in EPB41L3 expression in treated GOS3 and U87MG cells, respectively (Figure 2a). The treatment boosted the expression of TSP-1 in SW1783 and T98G cells. The level of RASSF2 expression was also higher in T98G and U87MG cells (*p* < 0.001), and most notably in SW1783 (an approximately 5-fold increase) (Supplementary Figure 1).

**Table 2:** Quantitative reverse-transcription PCR results in treated compared with control cell lines

Gene	Cell lines							
	GOS3		SW1783		T98G		U87MG	
	2 ^−ΔΔCt*^	P-*value*	2 ^−ΔΔCt^	P-*value*	2 ^−ΔΔCt^	P-*value*	2 ^−ΔΔCt^	P-*value*
*EPB41L3*	8.08	< 0.001	1.92	< 0.001	2.24	< 0.001	6.16	< 0.001
*RASSF2*	1.42	0.116	4.46	< 0.001	2.01	0.001	2.63	< 0.001
*TSP-1*	0.74	0.131	2.71	< 0.001	3.83	< 0.001	1.13	0.128

* relative change in expression of genes of the treated cells compared with the control group

### Analysis of EPB41L3, RASSF2 and TSP-1 expression by IF in control and treated cell lines and by IHC in tumors

IF exhibited a higher level of overall (total fluorescent content) RASSF2 expression in all treated cell lines, whereas overall EPB41L3 expression was higher in SW1783 cells, and TSP-1 expression was stronger in the overall content of treated T98G cells and in the cytoplasm of treated U87MG cells compared with controls (Figures 2b and 2c; Supplementary Table 1).

The expression of CD31, EPB41L3, RASSF2 and TSP-1 proteins in the tumoral tissue was analyzed by IHC. The level of p53 expression was less than 66% in most cases (Table 1, Supplementary Figure 2). The median number of microvessels detected by CD31 expression was 26.5 (range, 2–250). TSP-1 and p53 were expressed in the cytoplasm and nucleus of tumor cells, respectively, whereas EPB41L3 and RASSF2 staining was present in cytoplasm and/or nucleus.

EPB41L3, RASSF2 and TSP-1 expression was low or absent in the majority of cases with hypermethylated promoter (Figure 2d, Supplementary Figure 1d). Simultaneous nuclear and cytoplasmic EPB41L3 expression was found in 50% of the positive cases and was lost in 60% of the hypermethylated cases. TSP-1 expression was scored as intermediate–strong in 50% of the patients, and was reduced or absent in the other cases. The pattern of expression of this protein was heterogeneous in several hypermethylated cases, with different areas exhibiting strong and weak expression. Reduced TSP-1 staining with higher MVD was observed in several cases, although this association was not statistically significant (data not shown).

#### 1p/19q co-deletion

*1p* and *19q* deletions were present in 12 (33.3%) and 14 (38.9%) of 36 patients, respectively; both deletions were present in 9 cases (29%). This alteration was more frequent in cases non-methylated for EPB41L3 (41.2%) than in cases methylated for this gene (11.1%) (*p* = 0.114). This association was very clear in the case of the 19q deletion, which was present almost exclusively in cases non-methylated for *EPB41L3* (*p* = 0.014).

### Survival analysis

To determine whether any of these markers were of prognostic significance, the survival of all the patients, and those with astrocytic and oligoastrocytoma tumors, was analyzed. PFS and OS were significantly shorter in patients of advanced age and with higher tumor grade (Supplementary Tables 2 and 3). In the univariate analysis the *1p* and *19q* deletions and the *1p/19q* co-deletion were associated with longer PFS periods (*p* = 0.089, *p* = 0.014, and *p* = 0.008, respectively), but there were no differences in OS.

**Figure 3:**
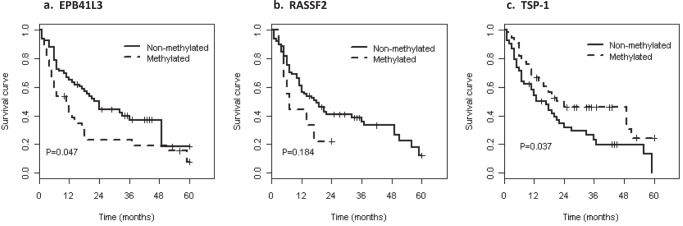
Kaplan–Meier curves predicting the probability of death in brain tumor patients, by methylation status

OS and PFS for the gene methylation (univariate analysis) are shown in Table 3, Figure 3 and Supplementary Figure 3. OS was shorter for patients with *EPB41L3* (*p* = 0.047) and without *TSP-1* hypermethylation (*p* = 0.037) (Figure 3). *RASSF2* hypermethylation was also a prognostic factor for poor outcome although the association was not statistically significant (*p* = 0.184). Similar results were obtained by the generalized Wilcoxon test, except for PFS in the case of *TSP-1* hypermethylation, for which there was marginal significance as demonstrated by the log-rank test.

**Table 3:** Univariate Kaplan-Meier analysis of the risk of progression or death related to pathological and molecular variables in patients with brain tumors

		*Progression*		*Overall survival*	
		*HR (95% CI)*	*p*	*HR (95% CI)*	*p*
***Total Sample (n = 132)***					
***EPB41L3* meth**	No	Ref.		Ref.	
	Yes	1.52 (0.91, 2.56)	0.109	1.68 (1.00, 2.82)	0.047
***RASSF2* meth**	No	Ref.			
	Yes	1.35 (0.48, 3.80)	0.572	1.71 (0.77, 3.80)	0.184
***TSP-1* meth**	No	Ref.		Ref.	
	Yes	0.66 (0.42, 1.04)	0.074	0.61 (0.38, 0.97)	0.037
***Astrocytic tumours (n = 103)***					
***EPB41L3* meth**	No	Ref.		Ref.	
	Yes	1.58 (0.87, 2.89)	0.130	2.45 (1.33, 4.49)	0.003
***RASSF2* meth**	No	Ref.		Ref.	
	Yes	1.52 (0.36, 6.47)	0.567	1.30 (0.46, 3.66)	0.616
***TSP-1* meth**	No	Ref.		Ref.	
	Yes	0.73 (0.45, 1.20)	0.210	0.72 (0.43, 1.21)	0.220
***Oligodendroglial tumours (n = 29)***					
***EPB41L3* meth**	No	Ref.		Ref.	
	Yes	2.11 (0.65, 6.82)	0.203	1.03 (0.37, 2.89)	0.959
***RASSF2* meth**	No	Ref.		Ref.	
	Yes	1.86 (0.34, 10.21)	0.467	4.63 (1.03, 20.86)	0.029
***TSP-1* meth**	No	Ref.		Ref.	
	Yes	0.52 (0.16, 1.69)	0.273	0.42 (0.14, 1.24)	0.107

CI, confidence interval; HR: hazard ratio; meth, methylation; Ref., reference value

After adjusting for age and tumor grade in the multivariate model (Table 4), the risk of progression and death remained significantly higher in patients with *EPB41L3* hypermethylation (HR = 1.79; 95% CI = 1.05, 3.05), but not for TSP-1 (HR = 0.8; 95% CI = 0.49, 1.31). According to *RASSF2* hypermethylation, the hazard ratio for survival was 2.16 (95% CI = 0.91, 5.1) after adjusting for age and tumor grade. In astrocytic and oligodendroglial tumors, *EPB41L3* hypermethylation was a prognostic factor of poor survival (*p* = 0.035), and *TSP-1* hypermethylation is a strong prognostic factor in oligodendroglioma, associated with prolonged survival (*p* = 0.003) (Table 4).

**Table 4:** Multivariate Cox proportional analysis for determining disease outcome based on the risk of death related to pathological and molecular variables, adjusted by age and tumor grade

*Total Sample*		*Progression*		*Overall survival*	
		*HR (95% CI)*	*p*	*HR (95% CI)*	*p*
***EPB41L3* meth**	No	Ref.		Ref.	
	Yes	1.45 (0.86, 2.46)	0.175	1.79 (1.05, 3.05)	0.035
***RASSF2* meth**	No	Ref.		Ref.	
	Yes	1.21(0.41, 3.55)	0.735	2.16 (0.91, 5.10)	0.102
***TSP-1* meth**	No	Ref.		Ref.	
	Yes	0.78 (0.49, 1.25)	0.304	0.80 (0.49, 1.31)	0.381
***Astrocytic tumors (n = 103)***					
***EPB41L3* meth**	No	Ref.		Ref.	
	Yes	1.33 (0.72, 2.45)	0.371	1.88 (1.01, 3.49)	0.046
***RASSF2* meth**	No	Ref.		Ref.	
	Yes	1.07 (0.25, 4.61)	0.266	1.95 (0.65, 5.89)	0.268
***TSP-1* meth**	No	Ref.		Ref.	
	Yes	0.94 (0.55, 1.60)	0.825	1.09 (0.63, 1.91)	0.751
***Oligodendroglial tumors (n = 29)***					
***EPB41L3* meth**	No	Ref.		Ref.	
	Yes	2.20 (0.64, 7.58)	0.209	1.21 (0.42, 3.47)	0.720
***RASSF2* meth**	No	Ref.		Ref.	
	Yes	1.88 (0.32, 11.22)	0.500	3.40 (0.69, 16.8)	0.149
***TSP-1* meth**	No	Ref.		Ref.	
	Yes	0.20 (0.04, 1.09)	0.056	0.11 (0.02, 0.58)	0.003

CI, confidence interval; HR: hazard ratio; meth, methylation; Ref., reference value

## DISCUSSION

GBM is the most frequent malignant glioma and is characterized by the highest grade of undifferentiation and aggressiveness. It is still a challenging cancer to treat, although therapy has improved in recent years [37]. The value of maximum feasible surgical resection of the tumor and the clinical assessment criteria for this approach remain subjects of debate [38]. In our study, we found a clearly increasing hazard ratio of disease progression from gross total resection to stereotactic biopsy. Age was also closely related with prognosis, as previously described [39].

Brain tumors are characterized by high molecular heterogeneity, which explains their differential responses to oncological treatment. In this study, we assessed the hypermethylation status of several genes known to be altered in other types of cancers. All of these genes are prone to becoming hypermethylated, which affects gene expression, as demonstrated here by qRT-PCR and immunofluorescence in the various cell lines treated with demethylating agents, in which reexpression varies according to nucleus and cytoplasm location. Importantly, we have described here for the first time the hypermethylation of *EPBB41L3* and *RASSF2* genes in tumors of astrocytic and oligodendroglial lineage. These hypermethylations were found to be independent of age, which is known to contribute decisively to the methylation of some other genes [39].

The role of the *RASSF2* gene in cancer remains largely unknown. Its cancer-suppressor role probably arises from its predominant expression in the cell nucleus and its function in growth inhibition [21]. The gene is silenced by hypermethylation in several types of non-nervous tumors, such as breast, lung and gastric cancer [21, 40] and as described for the first time by our group in cervical cancer [7]. It has also been found in nervous tumors, e.g., neuroblastoma, a type of childhood cancer that affects nerve tissue outside the central nervous system [23]. RASSF2 is also downregulated in malignant peripheral nerve sheath tumors (MPNSTs) associated with neurofibromatosis type 1 relative to non-malignant plexiform neurofibromas [41], probably as a consequence of its hypermethylation.

Our group reported for the first time that, in cervical and vulvar cancer, this gene is hypermethylated almost exclusively in tumors but not in benign lesions [7, 32, 33]. In this study, this alteration was present exclusively in tumors but not in non-pathological brain tissue, and was equally prevalent in all tumor grades. This suggests that the alteration is an early event, as occurs in colorectal cancer [42]. To our knowledge, this is the first description of *RASSF2* hypermethylation in astrocytic and oligodendroglial tumors. It is also of note that survival time in patients with this alteration was shorter than in patients with unmethylated tumors, although the difference was not statistically significant. The role of *RASSF2* hypermethylation in predicting poor outcome has been described in cervical and gastric cancer [7, 22, 40]. However, more studies with larger populations are needed to evaluate this alteration in brain tumors.

In our study, *TSP-1* hypermethylation was more frequent than *RASSF2* hypermethylation, and more frequent in less aggressive grade I-II tumors than in more advanced grades (III-IV) [37], which is evidence of the early presence of this alteration in brain tumors. It also appears to be a protective alteration, since patients with the hypermethylated promoter had longer survival periods than their counterparts with unmethylated promoter. This effect was evident in oligodendrogliomas. Previous studies reported a protective role for TSP-1, whereby it is an antiangiogenic endogenous factor that prevents tumor neovascularization [43], although with diverse functions in cancer progression. For example, TSP-1 promotes metastasis by up-regulation of the matrix metalloproteinases MMP-2 and MMP-9 [44]. TSP-1 expression predicts poor prognosis in high-grade gliomas [43], in accordance with the findings of the present study. As previously mentioned, *TSP-1* hypermethylation is clearly associated with a bad prognosis in oligodendrogliomas. Little is known about the involvement of TSP-1 in oligodendrogliomas except that it is mainly involved in the migration of oligodendrocyte precursor cells in normal central nervous system development [46].

A possible cause of the protective effect of *TSP-1* silencing by hypermethylation in GBM and OD is that TSP-1 is pro-migratory at the border of gliomas, as are other proteins secreted by glioma cells such as laminins, vitronectin and fibronectin [47]. These secreted proteins contribute to the invasion of brain tissue by glioma cells from preexisting brain structures [48], and have also been implicated in angiogenesis and synaptic recovery after brain injury [29]. An earlier study showed that tumors formed by the C6 glioma cells expressing the angiostatic peptide of TSP-1 (TSP1ang) strongly inhibited angiogenesis but became more aggressive and invasive [49]. Studies of these proteins will be crucial to assess the effectiveness of new antiangiogenic therapies by new radiographic techniques, particularly in relation to the use of bevacizumab in combination with radiation therapy. This has already yielded some encouraging preclinical results [50].

In our study, *EPB41L3* hypermethylation was observed in astrocytic and oligodendroglial tumors, and was preferentially present in oligodendroglial tumors that did not show 1p/19q co-deletion. It is also worth noting that *EPB41L3* hypermethylation takes place preferentially in advanced grades (III–IV) compared with early grades (I–II), pointing to the sequential involvement of epigenetic alterations, as previously described for other genes such as p14 [51].

The *EPB41L3* gene had the most striking prognostic role in hypermethylation in our study. The presence of this alteration was found to be related to metastasis in renal clear cell carcinoma [8]; the loss of expression was also described in lung cancer, in which it was also associated with metastasis [15]. The LOH of this gene was described in other brain tumors (ependymomas and meningiomas) [9, 12]; hypermethylation was suggested as being another mechanism for its inactivation in meningiomas [9]. Tumor allelic losses of the *EPB41L3* gene in ependymomas were reported to be associated with worse prognosis [52].

In the present study, hypermethylation of this gene was of prognostic significance in astrocytic and oligodendroglial tumors, particularly in GBM, suggesting that it has a role in this type of cancer.

In conclusion, to the best of our knowledge, this is the first time that *EPB41L3* and *RASSF2* hypermethylation has been observed in astrocytic and oligodendroglial tumors. It is also the first time that these alterations and TSP-1 hypermethylation have been shown to have different prognostic values. It will be important to test whether hypermethylation of these genes is related to other alterations such as IDH1 mutations and EGFR amplification found in brain tumors [53], Further studies will help clarify the usefulness of these alterations for detecting and managing patients with poor prognosis.

## MATERIALS AND METHODS

### Cell lines

Four previously authenticated human brain tumor cell lines (GOS-3, SW1783, T98G and U87MG) were studied (see *Supplementary Material*). The T98G and U87MG cell lines are derived from GBM (grade IV); GOS-3 and SW1783 cells are derived from mixed astrocytoma (grade III)/oligodendroglioma (grade II) and astrocytoma (grade III), respectively. These cells were grown in either Dulbecco's modified Eagle's medium (DMEM) or RPMI 1640 medium supplemented with 10% fetal bovine serum and 1% penicillin/streptomycin (Invitrogen, Life Technologies, Carlsbad, CA, USA). All cell lines were cultured in a humidified atmosphere at 37°C with 5% CO_2_.

### Patients

A cohort of 132 patients was studied. They had all been diagnosed with astrocytic and oligodendroglial tumors between 2000 and 2010 in the Pathology Department Section A of the Hospital Complex of Navarra (Navarra Public Health System). As neurosurgical resections are limited to tumors, no matched controls were available for the study, so 10 cases of normal brain tissue from healthy donors from the Neurological Tissue Bank of Navarra were included as controls. The study was approved by the Clinical Research Ethics Committee of Navarra.

Patients’ pathological and clinical data are summarized in Table 1. None of the patients had received radiation or chemotherapy before surgery and 20 of them (15.2%) had previous brain tumors.

The most frequent location of the tumors was frontal (50 cases, 38.2%), followed by temporal (28 tumors, 21.4%) and parietal (11 cases, 8.4%) locations. Diagnoses were confirmed following microscopic inspection by an experienced neuropathologist. In terms of tumor lineage 96 (73%) were astrocytic, 29 (22%) were oligodendroglial, and 7 (5%) patients were mixed astrocytic-oligodendroglial. The majority of cases (53%; 70 patients) were diagnosed with GBM (WHO grade IV), followed by anaplastic oligodendroglioma (WHO grade III) (19 patients), and oligodendroglioma (WHO grade II; 10 patients) and pilocytic astrocytoma (WHO grade I; 9 patients) (Supplementary Figure 2).

The oncological treatment of patients was administered according to standard protocols [31]. Adjuvant therapy was considered in 96 patients, whereas it was ruled out for 36 patients on the grounds of age, health status, and, in 8 patients, a diagnosis of astrocytoma grade I. The treatment consisted of radiotherapy alone (19 patients, 19.8%) or radiotherapy in combination with temozolomide (75 patients, 78.1%), or temozolomide alone (2 patients, 2.1%).

Patient follow-up included, in addition to physical and clinical examination, a radiological analysis by magnetic resonance every 3 months for high-grade tumors, and every 6 months for low-grade tumors.

Follow-up data were not available for 6 patients (4.5%); during the follow-up 76 patients (57.6%) died of the disease and 12 (9.1%) died of other causes. Currently, 29 (22.0%) and 9 (6.8%) patients are with and without illness, respectively.

### DNA extraction from cell lines and tissue

DNA was extracted from 0.5 × 10^6^ cells in the case of cell lines; a representative area with > 70% of tumoral cells from 5-μm-thick sections selected by the pathologist on formalin-fixed, paraffin-embedded sections stained with hematoxylin and eosin was considered for the tumors, as previously described [7]. Frozen tissue from normal cases was homogenized and digested with proteinase K and extracted by the phenol-chloroform method. DNA concentration and quality were measured using a NanoDrop spectrophotometer ND-1000 (Thermo Scientific, Hanover Park, IL, USA). To assess DNA quality, the ß-globin gene was amplified by PCR, as previously described [32].

### Methylation analysis

#### Methylation-specific PCR (MSP) in cell lines and tumors

MSP of *EPB41L3*, *RASSF2* and *TSP-1* genes was performed in all cell lines and cases after sodium bisulfite modification of 1 μg of genomic DNA. Modified DNA was amplified with previously described specific primers directed towards methylated and unmethylated sequences [7, 33], including positive and negative controls as previously described [7].

#### Confirmation of EPB41L3 and TSP-1 methylation by pyrosequencing

To verify the presence of DNA methylation of *EPB41L3* and *TSP-1* genes by a different method from MSP, pyrosequencing (PSQ) analyses were performed using bisulfite-treated DNA from 10 tumoral and 10 normal cases. PCR was performed with a 40-ng aliquot of modified DNA using primers, one of which (reverse) was biotinylated; after converting the PCR product to single-stranded DNA templates a specific sequencing primer was used (Supplementary Table 4). The set of primers for PCR amplification and PSQ was designed using a specific software package (PyroMark assay design version 2.0.01.15), ensuring methylation-independent amplification (Supplementary Table 4). These sets of primers included 6 and 13 CpGs in the *EPB41L3* and *TSP-1* genes, respectively. PSQ reactions and methylation quantification were performed in a PyroMark Q24 System version 2.0.6 (Qiagen, Heidelberg, Germany). The PSQ results were analyzed using PyroMark software (Qiagen) and converted into percentages of all CpGs showing methylation.

#### Confirmation of RASSF2 methylation by bisulfite sequencing

Bisulfite sequencing (BS) was used to confirm *RASSF2* methylation as it had been standardized for the analysis of this gene in cervical tumors, as first reported by our group [7]. In this technique, clones containing DNA previously analyzed by MSP from normal and methylated tumoral cases were sequenced using the 3100 Genetic Analyzer (Applied Biosystems, Life Technologies, CA, USA) after carrying out PCR of the fragment containing 6 CpG islands with specific primers (Supplementary Table 4). The results were converted into percentages of CpG islands in the methylated region analyzed.

### Expression analysis

#### 5-azadC and TSA treatment in cell lines

To explore the effect of DNA hypermethylation and histone acetylation on expression of *EPB41L3*, *RASSF2* and *TSP-1 in vitro*, cell lines were treated with demethylating and acetylating agents. Quantitative reverse-transcription PCR (qRT-PCR) and immunofluorescence (IF) were used to analyze mRNA levels and protein expression, respectively. Thus, GOS-3, LN18, T98G, and U87MG cell lines were seeded at low density for 24 h and then treated with 5 μg/ml 5-azadC for 96 h (Sigma, St. Louis, MO, USA) and 200 mg/ml TSA for the final 24 h (Sigma), both diluted in phosphate-buffered saline (PBS). The medium containing the drug was replaced every 24 h.

#### qRT-PCR for EPB41L3, RASSF2 and TSP-1 RNA expression in cell lines

RNA was isolated from control and treated cells with 5-azadC and TSA using the Ribopure kit including a *DNAse* I treatment (Ambion, Life Technologies, Carlsbad, CA, USA). Single-stranded cDNA from 1 μg of RNA obtained was synthesized using the Reverse Transcription kit (Life Technologies). Real-time PCR reactions for analyzing the expression of the three genes were carried out in triplicate for each gene and each case using 100 ng of cDNA with TaqMan Universal PCR Master Mix (Life Technologies), 1x TaqMan gene expression assays (EPB41L3; Assay Id: Hs00202360_m1; RASSF2: Hs00248129_m1; TSP-1; Assay Id: Hs00962908_m1; Life Technologies) and GAPDH as an endogenous control, as previously reported [7]. The relative change in the expression of treatment genes compared with the control value was calculated by the delta-delta Ct method.

#### Immunofluorescent detection of EPB41L3, RASSF2 and TSP-1 expression in cell lines

To determine whether demethylating treatment restores protein expression immunofluorescence staining was performed in treated and control cell lines, and fluorescence intensity and location of the proteins (nuclear and cytoplasmic) were analyzed. After treatment, cells were fixed in fresh 4% paraformaldehyde (Merck, Darmstadt, Germany), permeabilized with 0.5% Triton 100-X for 30 min at room temperature, and blocked with 10% fetal bovine serum in PBS for 2 h. Subsequently, fixed cells were incubated with primary antibody against EPB41L3 (mouse, 1:150; Sigma, St. Louis, MO, USA), RASSF2 (rabbit, 1:150; Epitomics, Burlingame, CA, USA) or TSP-1 (mouse, 1:150; Everest Biotech, Ltd., Upper Heyford, UK) at 4°C overnight. Slides were then incubated with Alexa Fluor 488-conjugated secondary antibody (goat anti-mouse IgG, 1:200, Life Technologies, for EPB41L3; goat anti-rabbit IgG, 1:200, for RASSF2; Life Technologies). Phalloidin (Alexa Fluor 594, 1:500; Invitrogen) and DAPI counterstain (Abbott Molecular, Abbott Park, IL, USA) were added for nucleus and cytoplasm detection. Confocal microscopy was performed with a Leica TCS SP5 laser scanning microscope (AOBS) (Leica, Wetzlar, Germany) sequentially using excitation wavelengths of 488 nm (for FITC) and 561 nm (for Texas Red). To measure fluorescence intensities of nuclear and cytoplasmic EPB41L3, RASSF2 and TSP-1, at least 100 cells from each condition group were examined using a 63× HCX PL APO CS oil immersion objective 1.4 (NA). The average fluorescence intensities of nuclei and cytoplasm, and the nucleo-cytoplasmic ratios were quantified with Definiens XD Software (Definiens, Munich, Germany) and pooled for each condition.

#### Immunohistochemistry studies in tumors

Given the value of Ki-67 and p53 as markers of malignancy [1], they were immunohistochemically characterized in all the tumors, whereas EPB41L3, RASSF2, TSP-1 and CD31 proteins were examined in a sample of tumors with methylation results to establish whether there was an association between hypermethylation and reduced expression. Thus, EPB41L3 and TSP-1 were evaluated in 20 patients (10 GBM and 10 OD; 5 hypermethylated and 5 unmethylated cases per group). RASSF2 was evaluated in the 10 hypermethylated cases (grades II, III and IV) and in 10 additional unmethylated cases. For this technique, 4-μm-thick sections of tumors were mounted on glass slides, deparaffinized and rehydrated. Slides were then incubated with antibodies against the aforementioned markers after antigen retrieval with Bond Epitope Retrieval Solution (Leica Biosystems Newcastle Ltd., Newcastle upon Tyne, UK) under the conditions listed in Supplementary Table 5. Primary antibodies were developed using Bond Polymer Refine Detection (Leica Biosystems Newcastle Ltd.) and visualized with DAB (3, 3′-diaminobenzidine). Finally, slides were counterstained with hematoxylin. The extent and intensity of immunopositivity were both considered, and immunohistochemistry (IHC) was independently evaluated by two experienced neuropathologists who had no prior information about the cases. Ki-67 expression was scored for low, intermediate and high proliferation indices, and CD31 was evaluated to calculate microvessel density (MVD) in accordance with previous criteria in the same cases in which TSP-1 had been evaluated [32].

The nuclear and/or cytoplasmic intensities of the other proteins were ascribed to one of four categories: 0, negative (no positive cells); 1, weak (< 30% positive cells); 2, moderate (31–60% positive cells); 3, strong (> 60% positive cells), as previously described [7, 32].

### Analysis of deletion 1p/19q in oligodendrogliomas by FISH

To better characterize oligodendroglial tumors and astrocytic tumors with OD differentiation (36 cases) the losses in *1p36* and *19q.13.3* were analyzed by fluorescence *in situ* hybridization (FISH), using the mixture of LSI *1p36 SpectrumOrange* and *1q25 SpectrumGreen* probes for *1p* deletion and the mixture of *19q13 SpectrumOrange* and *19p13 SpectrumGreen* probes for *19q* deletion (Vysis, New York City, NY, USA), as previously reported [34].

### Statistical analysis

Demographic, clinical and pathological data were summarized as means (and standard deviations, SDs) or frequencies (and percentages), as appropriate. Group means for *EPB41L3* and *TSP-1*, obtained from CpG sites by PSQ in tumoral and normal cases, were compared using the Mann–Whitney U test. The presence of methylation of *RASSF2* in clones derived from each tumoral and normal sample was assessed graphically. Associations between the percentage of methylation and patient age were measured by the Spearman coefficient.

Associations between gene hypermethylation, pathological and clinical variables of this retrospective study were assessed by the X^2^ or Fisher's exact test; the X^2^ test was used to examine differences in proportions, and associations between methylation of MSP, PSQ (*EPB41L3*, *TSP-1*) and BS (*RASSF2*). Differences in levels of expression detected by qRT-PCR between control and treated cell lines were evaluated by two-tailed unpaired Student's t tests.

To evaluate IF results, the mean values for each condition (control *vs*. treated) were compared using two-tailed unpaired *t*-tests, stratified by location (nucleus or cytoplasm), considering the sum of the values for the two compartments.

Finally, the times between the date of surgery and the date of recurrence or death were used to estimate, respectively, progression-free survival (PFS) and overall survival (OS). Kaplan–Meier plots and the log-rank test were used to examine the differences in survival time between patients with methylation-positive and methylation-negative tumors. These analyses were complemented by the generalization of the Wilcoxon–Mann–Whitney score for interval-censored data. Cox proportional hazard regression models were used in a multivariate setting to test the independent contribution of each variable to the outcome after adjusting for other potential confounders. Hazard ratios (HRs) and 95% confidence intervals were used to estimate the effect of each variable on the outcome.

## SUPPLEMENTARY FIGURES AND TABLES


