# Supplementary material for: EPB41L3, TSP-1 and RASSF2 as new clinically relevant prognostic biomarkers in diffuse gliomas
Source: Oncotarget. 2015 Jan 30;6(1):368–80. doi: 10.18632/oncotarget.2745 (PMC4381601; doi:10.18632/oncotarget.2745)
Supplement: Supplementary file 1 [file oncotarget-06-368-s001.pdf]

SUPPLEMENTARY FIGURES AND TABLES

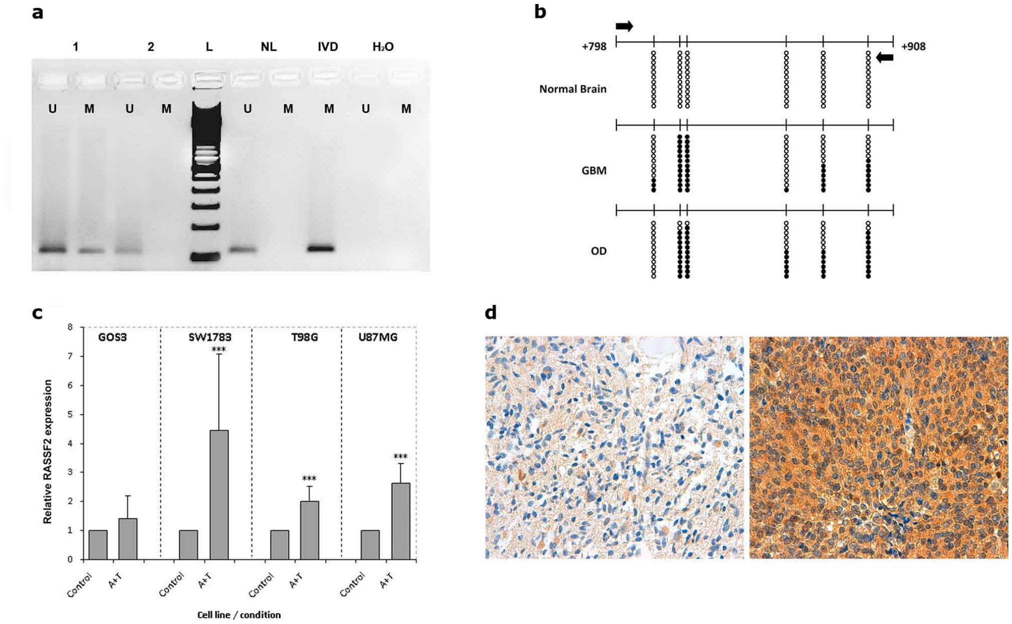

Supplementary Figure 1: (a) MSP image of RASSF2 methylation analysis. Unmethylated (U) and methylated (M) PCR products are shown for each case. (NL: DNA from normal lymphocytes; IVD: in vitro-methylated DNA). **(b)** Bisulfite-sequencing analysis of RASSF2 promoter methylation status in 12 clones containing the RASSF2 sequence from cases of normal brain tissue, GBM and OD. The methylation status of analyzed CpG sites is shown for each clone (open and filled circles represent unmethylated and methylated clones, respectively). Black arrows indicate the location of the MSP primers. The location of each CpG site relative to the transcription initiation site is shown by a vertical bar. **(c)** Expression level of RASSF2 in brain tumor cell lines treated with 5-azadC and trichostatin. The relative level of expression after qRT-PCR was compared with the untreated cells (Ctl), whose value was taken as 100%. Bars represent the mean ± standard deviation of the relative expression level of three experiments. *** indicate statistically highly significant ($p<0.01$) differences in gene expression between treated and untreated cells. **(d)** IHC staining for a tumor positive (left) and negative or with weak expression of RASSF2 (right), unmethylated and methylated for this gene, respectively.

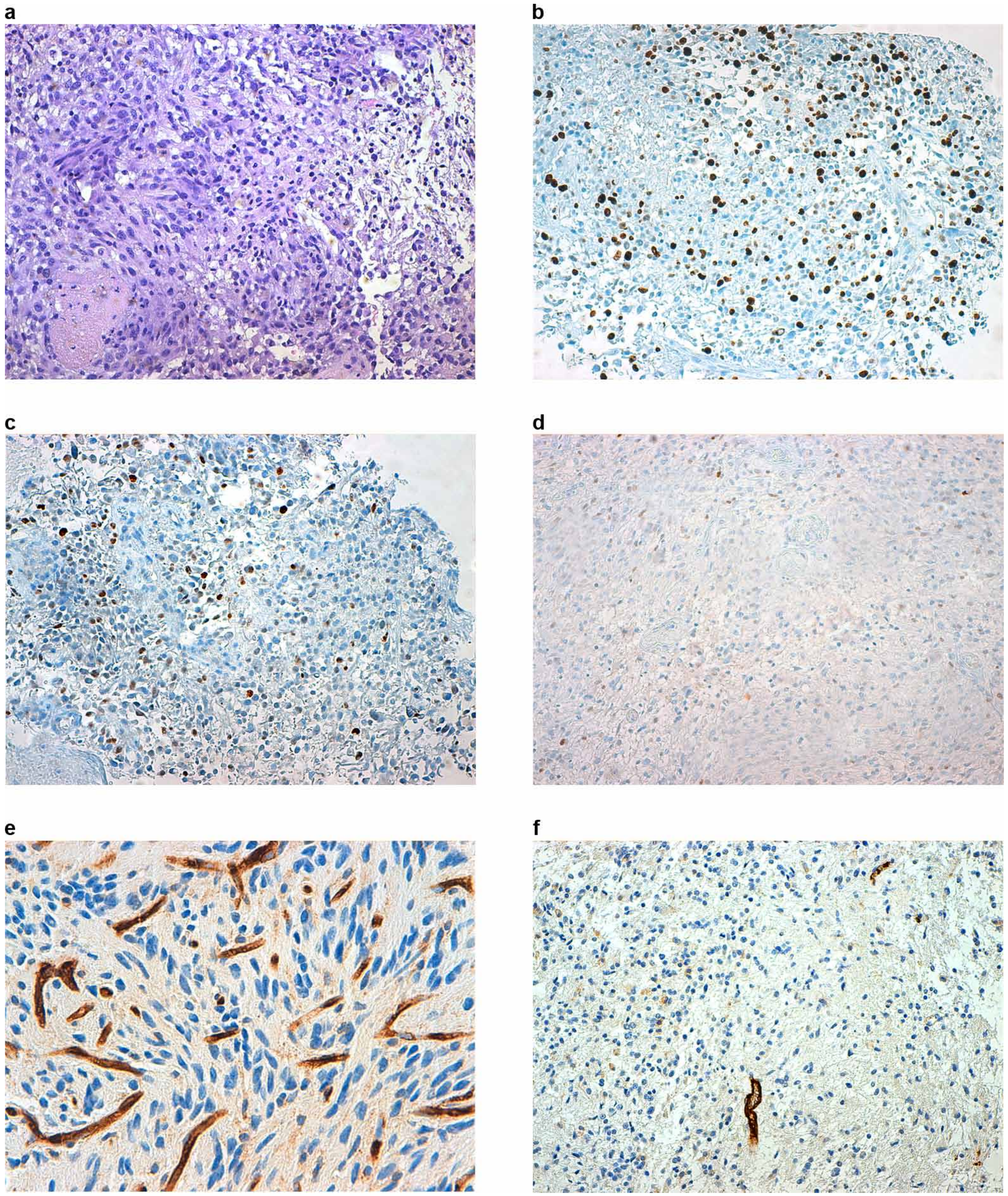

Supplementary Figure 2: (a) Microscopic view of a GBM (hematoxylin and eosin, x200). (b) Ki-67 immunohistochemistry (IHC) for a highly proliferative GBM (x200). **(c)** A case of GBM with p53 IHC expression in 70% of cells (x200). **(d)** A case of GBM with p53 IHC expression in 40% of cells (x200). High-density **(e)** and low-density **(f)** neoplastic vessels in the tumor are highlighted by CD31 staining (×400 and x200, respectively).

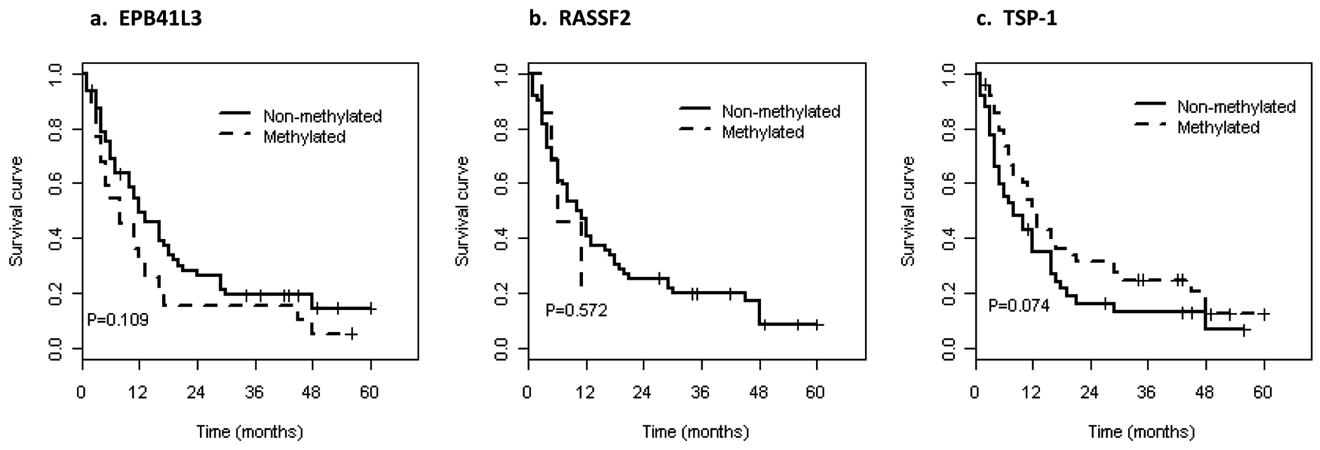

Supplementary Figure 3: **Kaplan–Meier curves predicting the probability of progression in brain tumor patients, by methylation status.**

Supplementary Table 1: Immunofluorescence values in treated cell lines compared with control cell lines

	Total		Cytoplasm		Nucleus	
**Cell line / Gene**	**ΔTx *vs* Ctl (95% CI)**	**p**	**ΔTx *vs* Ctl (95% CI)**	**p**	**ΔTx *vs* Ctl (95% CI)**	**p**
**GOS3**						
EPB41L3	–4.84 (–6.34, –3.35)	< 0.001	–2.26 (–3.82, –0.70)	0.005	–7.42 (–9.95, –4.90)	< 0.001
RASSF2	6.13 (5.61, 6.66)	< 0.001	2.20 (1.87, 2.54)	< 0.001	10.06 (9.34, 10.78)	< 0.001
TSP-1	–24.58 (–28.20, –20.96)	< 0.001	4.21 (1.41, 7.02)	0.003	–54.8 (–59.3, 50.21)	< 0.001
**SW1783**						
EPB41L3	1.03 (0.18, 2.23)	0.094	1.28 (0.72, 1.83)	< 0.001	0.78 (–1.59, 3.14)	0.515
RASSF2	1.86 (0.46, 3.27)	0.010	2.42 (0.21, 4.63)	0.032	1.30 (–0.45, 3.05)	0.145
TSP-1	–5.67 (1.09, 2.22)	< 0.001	2.85 (2.04, 3.01)	< 0.001	–4.95 (–1.23, –4.78)	0.002
**T98G**						
EPB41L3	–7.96 (–9.20, 6.72)	< 0.001	–0.17 (–0.82, 0.48)	0.604	–15.75 (–17.75, 13.76)	< 0.001
RASSF2	1.55 (1.19, 1.90)	< 0.001	1.26 (0.94, 1.58)	< 0.001	1.82 (1.19, 2.46)	< 0.001
TSP-1	1.66 (1.09, 2.22)	< 0.001	2.05 (1.24, 2.86)	< 0.001	1.26 (0.47, 2.05)	0.002
**U87MG**						
EPB41L3	–4.76 (–6.08, 3.44)	< 0.001	–3.14 (–3.95, 2.33)	< 0.001	–6.39 (–8.89, 3.88)	< 0.001
RASSF2	1.53 (1.06, 2.00)	< 0.001	0.60 (0.26, 0.94)	0.001	2.46 (1.59, 3.33)	< 0.001
TSP-1	–0.62 (–0.84, –0.41)	< 0.001	–0.22 (–0.59, 0.14)	0.228	–0.98 (–1.21, –0.75)	< 0.001

Δ Tx *vs* Ctl, IF intensity of treated cell lines *versus* IF intensity of control cell lines.

Supplementary Table 2: Univariate Kaplan –Meier analysis of the risk of recurrence or death related to demographic and pathological variables in patients with brain tumours

		Progression		*Overall survival*	
		HR (95% CI)	**p**	***HR (95% CI)***	**p**
**Age**		1.02 (1.00, 1.04)	0.012	1.04 (1.02, 1.06)	< 0.001
**Resection grade**	Total	Ref.		Ref.	
	Subtotal	0.66 (0.37, 1.16)	0.120	1.05 (0.57, 1.92)	0.235
	Partial resection	0.86 (0.40, 1.84)		0.93 (0.39, 2.22)	
	Stereotactic biopsy	1.32 (0.65, 2.68)		1.89 (0.88, 4.03)	
**Tumour grade**	I	Ref.		Ref.	
	II	2.85 (0.76, 10.74)	0.003	2.79 (0.58, 13.47)	< 0.001
	III	3.16 (0.93, 10.77)		3.20 (0.73, 14.12)	
	IV	5.51 (1.72, 17.70)		8.38 (2.04, 34.50)	
**Tumor lineage**	Oligodendroglial	Ref.		Ref.	
	Glial	1.64 (0.94, 2.84)	0.076	1.33 (0.79, 2.24)	0.277
**Tumour type**	Primary	Ref.		Ref.	
	Secondary	0.50 (0.28, 0.90)	0.018	0.75 (0.43, 1.29)	0.299

Supplementary Table 3: Models obtained by multivariate Cox proportional analysis of the risk of progression or death related to pathological and molecular variables in patients with brain tumours

		Progression		*Overall survival*	
		HR (95%CI)	***p***	***HR (95%CI)***	***p***
**M_1: Model for *TSP-1***					
**Age**		1.02(1.00, 1.04)	0.014	1.04(1.01, 1.06)	< 0.001
**Tumour grade**	**I**	Ref.		Ref.	
	**II**	3.25(0.83, 12.69)		2.02(0.40, 10.18)	
	**III**	3.40(0.97, 11.83)	0.004	3.14(0.70, 14.2)	< 0.001
	**IV**	5.19(1.60, 16.85)		5.95(1.42, 24.9)	
***TSP-1* meth**	**No**	Ref.		Ref.	
	**Yes**	0.78(0.49, 1.25)	0.304	0.80(0.49, 1.31)	0.381
**M_2: Model for *EPB41L3***					
**Age**		1.01(0.99, 1.03)	0.073	1.04(1.02, 1.06)	< 0.001
**Tumour grade**	**I**	Ref.		Ref.	
	**II**	1.69(0.41, 6.91)		1.10(0.21, 5.84)	
	**III**	2.41(0.68, 8.46)	0.076	1.89(0.41, 8.73)	0.001
	**IV**	3.12(0.95, 10.28)		3.64(0.88, 15.45)	
***EPB41L3* meth**	**No**	Ref.		Ref.	
	**Yes**	1.45(0.86, 2.46)	0.175	1.79(1.05, 3.05)	0.035
**M_3: Model for *RASSF2***					
**Age**		1.02(0.99, 1.04)	0.034	1.03(1.01, 1.06)	< 0.001
**Tumour grade**	**I**	Ref.		Ref.	
	**II**	5.86(0.68, 50.45)		2.88(0.33, 25.11)	
	**III**	7.92(1.03, 60.65)	0.002	4.32(0.56, 33.44)	0.010
	**IV**	12.24(1.66, 90.45)		8.63(1.15,64.77)	
***RASSF2* meth**	**No**	Ref.		Ref.	
	**Yes**	1.21(0.41, 3.55)	0.735	2.16(0.91, 5.10)	0.102

Supplementary Table 4: Primers used for pyrosequencing (EPB41L3 and TSP-1) and bisulphite sequencing (RASSF2) .

Pyrosequencing			
Gene	*PCR_Sense primer (5′-3′)*	*PCR_Antisense primer (5′-3′)-Biotin labelled*	*Pyrosequencing primer (5′-3′)*
EPB41L3	GGAGGTTGTGTTTATGTAATTGT	CCCCCTATTACAAAAAACAC	GTGTTTATGTAATTGTTTTGAAG
TSP-1	GGGTTTTTTTAGGTGGTTTT	AAACCAATCTAAACTCCTCTCT	GGGATGTTTGTTGATTAT
Bisulphite Sequencing			
Gene	*Sense primer (5′-3′)*	*Antisense primer (5′-3′)*	
RASSF2	TTTGTTGGAGTGTTTTGTTTTT	CTTTTAAACCCCCAAAATTTC	

Supplementary Table 5: Panel of primary antibodies and conditions used in IHC

Marker	*Clone*	*Type*	*Pretreatment (pH, T, duration)*	*Dilution*	*T, incubation time*	*Commercial source*
**Ki-67**	30–9	monoclonal	8.4, RT, 15 min	pre-diluted	37°C, 20 min	Roche (Mannheim, Germany)
**P53**	Bp53–11	monoclonal	8.4, RT, 15 min	pre-diluted	37°C, 36 min	Roche (Mannheim, Germany)
**CD31**	JC70A	monoclonal	9.0, 96°C, 15 min	1:30	RT, 20 min	Dako (Glostrup, Denmark)
**EPB41L3**	2B1	monoclonal	6.0, 96°C, 20 min	1:750	RT, 20 min	Sigma Aldrich (Steinheim, Germany)
**RASSF2**	EPR6621	monoclonal	9.0, 95°C, 30 min	1:100	RT, 60 min	Epitomics (San Diego, CA, USA)
**TSP-1**	A6.1	monoclonal	6.0, 96°C, 20 min	1:50	RT, 20 min	Pierce Thermo Scientific (Rockford, IL, USA)

min: minutes; RT: room temperature; T: temperature
